# Effects of Dietary Zn/Se and α-Tocopherol Supplementation on Metabolic Milieu, Haemogram and Semen Traits of Breeding Stallions

**DOI:** 10.1007/s12011-020-02447-7

**Published:** 2020-10-23

**Authors:** Maria Grazia Cappai, Andrea Taras, Ignazio Cossu, Raffaele Cherchi, Corrado Dimauro, Francesca Accioni, Gianpiero Boatto, Mario Deroma, Emanuela Spanu, Domenico Gatta, Cecilia Dall’Aglio, Walter Pinna

**Affiliations:** 1 Department of Veterinary Medicine, University of Sassari, Via Vienna No. 2, 07100 Sassari, Italy; 2 Department of Equine Breeding and Reproduction Research, Autonomous Region of Sardinia, 4th of Lucrezia Borgia Square, 07040 Ozieri, Italy; 3 Department of Agriculture, University of Sassari, viale Italia no 39, 07100 Sassari, Italy; 4 Department of Pharmacy and Chemistry, University of Sassari, Via Muroni No. 23, 07100 Sassari, Italy; 5 Laboratory of Mineralogy, Department of Agriculture, University of Sassari, Viale Italia No. 39, 07100 Sassari, Italy; 6 Department of Veterinary Sciences, University of Pisa, Via delle Piagge No. 2, 56124 Pisa, Italy; 7 Department of Veterinary Medicine, University of Perugia, Via San Costanzo No. 4, 06126 Perugia, Italy

**Keywords:** Antioxidant, Horse, Supplement, Trace elements, Vitamin E group

## Abstract

Trace element status and metabolic milieu are sometimes overlooked in common veterinary clinical practice across animal species. The evaluation of requirements of trace elements, in fact, may be useful to prevent the perturbation of tissue-specific metabolic impair. In particular, essential trace elements in the diet play key roles within sub-cellular metabolic patterns with macro effects at the systemic level, like blood cell stability and semen quality. This effect was studied in breeding stallions, in which semen quality and haemogram are important for reproduction. A case-control feeding trial involved 40 stallions (age: 8–21 years; body weight, BW: 510–531 kg) of one stud centre, allotted to two experimental groups (*n* = 20 control, CON vs. *n* = 20 supplemented, SUPPL100), following a matched-pairs approach based on age. Supplemented stallions (SUPPL100) received a mixed mineral and vitamin supplement of Zn/Se and α-tocopherol (α-TOH) (100 g/day stallion) to compound feed, fed as control diet to horses of the control group (CON). Horses resulted deficient in circulating α-TOH and Zn at the start, though clinically healthy. After supplementation, different plasmatic levels of α-TOH, Zn and Se were found between groups. Circulating basophils (BASO) and mean cell haemoglobin concentration (MCHC) were affected by the dietary treatment (*p* < 0.05). Plasmatic Se affected monocyte count, haematocrit, mean cell volume and mean cell haemoglobin concentration. Semen traits were not affected by the dietary treatment per se, except for mobile/progressive sperm cells (%) of stallions aged > 13 years marginal circulating levels of α-TOH (*p* = 0.04). Ameliorating the micromineral status showed to improve the haemogram of stallions in view of circulating levels of Cu. Semen quality appeared to be strongly dependent on animal effects.

## Introduction

Trace element status and metabolic milieu are sometimes overlooked in common veterinary practice across animal species. The evaluation of animal requirements of micronutrients, following a detailed evaluation of physiological stage and level of activity, should be useful to prevent the perturbation of tissue-specific metabolic impair. The onset of disorders is in general clinically manifest on a long-term period of inadequate daily intake. In particular, some trace elements play several key roles within sub-cellular biochemical patterns with macro effects at the systemic level. Functional needs normally exceed tissue maintenance conditions. In the specific case of not fulfilment of metabolic demands, chronic undersupply may be the reason for poor performance or disorders. However, it should also be emphasized that some trace elements display a narrow safety range, due to toxic effects in case of excessive intake. Thus, trace element status and metabolic milieu should be consciously evaluated to avoid unneeded supplementation and therefore tailored to animal’s needs.

In the stud horse, semen quality and haemogram are important for reproduction. Blood and sperm cells appear to be among those cellular lines markedly susceptible to chronic trace element deficiency, probably also due to the easiness of tissue sample for diagnostic exploration. A number of scientific papers [3, 8–10, 21, 26, 29, 37, 41, 44] report the effects of different groups of biologically active substances in synergy with trace elements with impacts on the metabolism of blood cells and spermatozoa in vitro (semen quality preservation both on fresh or thawed semen doses). However, limited data is reported from experimental feeding trials on effective nutritional supplementation in view of a detailed assessment of the metabolic milieu, with specific regard to micronutrient status (trace elements and vitamins) and the evaluation of requirements. However, in this regard, different results about the dietary modulation of semen quality are reported in literature and do not seem to converge to a consensus. In fact, the intake of different classes of nutrients with specific biological properties is reported to impact on some semen features to different extents [3, 9, 10, 37]. The interaction between dietary micronutrients and endogenous biochemical pathways of animals is well established and the adequate evaluation of potential supplementation should be considered in view of the orchestration from synergistic biological activities of macro-minerals, trace elements and vitamins as a whole. Among these, dietary antioxidants may play a supportive role, given the high metabolic activity of blood and sperm cells turn over and susceptibility to oxidative stress. Nutrients with antioxidant properties can serve as scavengers of reactive oxygen species (ROS) which are side products of the metabolic activities at sub-cellular levels. Blood and sperm cells are rapidly susceptible to such changes, due to the peculiar metabolism and interaction across the cell membrane between intra- and extra-cellular environments.

Stud stallions are horses with high genealogical value and brilliant sportive career. They are retired from horse racing and are lifelong reared for reproduction. Enrolment of stallions in the studbook is commonly based on the genetic potentials accompanied to a desired phenotype to fit equestrian disciplines, thereby determining the economic value of the semen. However, sports achievements do not necessarily mirror reproductive attitudes of the stallion [29, 38, 40]. Recently, different systems for the selection of breeding stallions have started to consider the quality of semen as a prerequisite to be enrolled in the stud season. With the establishment of standards for morphological and quantitative traits of fresh semen (like the percentage of immobile sperm cells and morphological characteristics of mobile and progressive spermatozoa) stallions can enter the stud season if matching with minimum requirements. It is well known that semen quality is not a synonym of fertility [8, 21, 26, 41], but some semen characteristics may represent a reliable start point to estimate the reproduction potentials of the stallion. This being said, several exogenous and endogenous factors are accounted to contribute to semen quality [22, 35].

Dietary antioxidants like selenium (Se) in association with α-TOH are known to support endogenous antioxidant systems to reduce ROS damage. Se and vitamin E [12] synergistically take part in several biochemical pathways and metabolic processes in the animal body. Se in the horse is characterized by a very narrow safety range [16, 19, 27, 31]. Plants can convert Se from the soil into Se-Methionine (organic form), which is the form that can be more efficiently retained and utilized in the animal body [4, 13]. α-TOH is synthesized and stored chiefly in the foliage of green plants. The antioxidant effect of Se is mainly linked to its presence in enzymatic proteins such as the glutathione peroxidase (GPX) family and as co-enzyme, in superoxide dismutase (SOD) groups [13, 39]. Zinc can take part in different metabolic activities as well, in particular important for tegument and muscle tissues, often acting in synergy with vitamin A.

Copper is of fundamental importance for the sub-cellular metabolism. In the horse, as well in other animal species, Cu is involved in the synthesis of haemoglobin, keratin synthesis, myelination of neurons or bone formation. In addition, Cu takes part in several enzymatic and antioxidant endogenous systems [18, 28].

It was hypothesized that beneficial effects on semen traits may be achieved through adequate assessment of selected micromineral circulating levels to tailor the supply of α-TOH, Zn, Cu and Se and in the diet of breeding stallions. In regard to this, the supplementation should meet the requirements of the horse also in view of individual metabolic milieu. Provision of such nutrients follows the recommendations to comply with the safety range, especially for Se and Cu due to toxic effects. The present investigation was undertaken with the aim to explore the effect of a combination of α-TOH, Zn and Se-Meth in the diet of breeding stallions on overall metabolic status to explore the effect on haematological profile and semen traits.

## Materials and Methods

### Animal Care

Animal handling complied with the recommendations of European Union Directive 2010/63/EU concerning animal care and further Consolidated Commission Implemented Decisions 2012/707/EU and 2104/11/EU. All procedures reported in this trial belong to conventional veterinary practices; in particular, blood and semen sampling were carried out by veterinary surgeons and trained technicians working in the stud farm of the Autonomous Region of Sardinia in compliance with good veterinary practices. Animals enrolled underwent conventional sampling for health monitoring as scheduled every 2 months according to protocols in force in the stud centre. Specific analyses on the same blood and semen samples were carried out in this trial, following the experimental feeding.

### Experimental Design

A total of 40 stallions (body weight: 510–531 kg; age: 8 to 21 years old) underwent an experimental case-control feeding trial for 8 weeks. All animals involved in the study served as breeding stallions in the previous stud season (January to June 2018). Inclusion criteria of animals in the trial took into account (a) serving frequency during the previous stud season (three times a week, on alternate days) and (b) healthy conditions (assessed by veterinary physical inspection and haematological, biochemical and serological analyses) and compliance with mandatory vaccinations for the prevention of infectious diseases.

The experimental feeding matched with the negative photoperiod of the boreal hemisphere, during the months of October and November 2018. The 40 stallions were allotted to two groups, following a matched-pairs approach based on age, consisting of 20 stallions each (median age of groups: 13 years). One group (SUPPL100) switched to the supplemented diet, in which additional all-rac-tocopheryl-acetate (α-TOH), Zn hydrate and seleno-methionine (Se-meth) in granular form were mixed with the compound pelleted feed administered in the morning meal. The other group continued to be fed with the basic diet and represented the control group (CON). Both groups received the same hay. Water was supplied ad libitum*.*

### Dietary Regimen and Feeding Practices

Rationing was based on hay and compound feed, by estimating a daily capacity of kg of feed DM intake up to a 2.5% of horse BW, subdivided into 1.1% from hay and 0.9% from the compound feed. Daily administration of hay was scheduled at 6:00 am and at 2:00 pm. The chemical composition of hay is reported in Table 1. Compound feed was in the form of a pelleted diet. Daily administration of compound feed was scheduled at 11:00 am and at 5:00 pm, offered on a daily basis. The granular supplement was mixed with the compound of SUPPL100 stallions, at an amount of 100 g/500 kg BW/horse, as fed. The supplement was mixed daily in the morning meal and provided additional all-rac-α-tocopherol (α-TOH), Se-methionine (Se-Meth) and zinc hydrate. The chemical composition of pelleted feed and granular supplement is reported in Table 1. During the last week of experimental feeding, individual daily intake of compound feed was calculated by weighing feed leftover around the 24 h.

**Table 1:** Ingredients and chemical composition of offered diets

Item^2^	Diet^1^	
	CON	SUPPL100
Ingredient (kg/day per horse, as fed)		
Hay^3^	10.0	10.0
Compound feed	4.50	4.50
Supplement^4^	-	0.10
Chemical composition of compound feed (g/kg feed)		
DM	881	881
NDF	553	553
CP	140	141
Ash	135	135
Ether extract	40.5	39.3
Zn (mg/kg)	120	122
Cu (mg/kg)	40.0	39.3
Se (mg/kg)	0.25	0.25
α-TOH (mg/kg)	44.1	221

^1^Diet: *CON*, control diet; *SUPPL100*, diet containing 100 g/day per horse of supplement

^2^Item: *DM*, dry matter; *NDF*, neutral detergent fibre; *CP*, crude protein; *α-TOH*, alpha-tocopherol

^3^Hay (oat/ryegrass/clover based) chemical composition: DM = 88.7%; CP = 12.9% of DM; NDF = 58.5% of DM; ADF = 34.2% of DM; ash = 13.5% of DM; ether extract = 2.16% of DM

^4^Supplement, chemical composition: DM = 90.1%; CP = 22.3%; ash = 12.1%; ether extract = 31.0%; zinc hydrate = 0.25%; seleno-methionine = 0.002%; all-rac-α-tocopheryl-acetate 1%

Recommendations [31]: zinc, 1 mg/kg BW; copper, 0.1 mg/kg BW; selenium, 2.5 μg/kg BW; α-TOH, 1 g/500 kg BW

### Blood and Semen Sampling Schedule

At the beginning and end of the feeding trial (*T*_0_ and *T*_1_) all horses were sampled for whole blood and semen, according to the protocols for health monitoring of horses in the stud centre. Specific analyses of target parameters were carried in the laboratory of the Division of Animal Nutrition of the Department of Veterinary Medicine and the laboratory of Biological Chemistry of the Department of Chemistry and Pharmacy of the University of Sassari. All animals were sampled for semen at *T*_0_ and *T*_1_ within 0, 24 and 48 h of blood sampling, to minimize the effect of immobile spermatozoa from random sampling during non-stud season. In addition, each horse was clinically inspected and assessed for body condition scoring by 9 points-scale [20].

### Laboratory Analyses of Feeds, Whole Blood, Blood Serum and Semen

Feeds and leftovers were analysed for nutrient composition. Samples were oven-dried (103 °C) and then ground (0.5 mm) in duplicate and analysed according to Weende analysis described by Naumann and Bassler [32]. The crude protein content was determined using the Dumas combustion method. The ether extract was determined by the Soxhlet apparatus. Fibrous fractions were determined according to Van Soest method [42].

Blood samples were taken at start and end of the feeding trial (*T*_0_ and *T*_1_) complete haematological and biochemical profile for the purpose of the trial were carried out. Sampling was scheduled at 8:00 am and whole blood was collected from the jugular vein into 9.0 mL vacuum collection whole blood tube with spray-coated K_2_EDTA (Vacutainer Systems Europe; Becton Dickinson, Meylan Cedex, France). Haematological profile was determined through an automatic analyser (Mindray BC-5000 Vet, Alcyon, Italy) and interpreted in view of own reference intervals.

Gel tubes with a clotting accelerator were also used for blood serum analyses. All blood samples were stored and refrigerated during the transport to the laboratory and processed within 6 h of sampling. Tube samples were centrifuged at 1500*g*, at for 4 °C for 10 min. To preserve samples from light, tubes were singly enveloped by tin foils. Plasma and serum of each horse were removed and stored in different aliquots in vials (2 mL) and frozen at − 20 °C until analysis, without exposure to light to minimize the effect due to photolability of α-TOH.

One aliquot was used to determine plasmatic concentrations of circulating Cu, Se, Zn and α-TOH. The rest of biochemical parameters, including nutrient-related metabolites, enzymes of organ function and elements were determined on serum within 1 week of sampling and processed by an automatic biochemical analyser (Mindray BS-200, Alcyon, Italy). Investigated parameters included: nutrient-related metabolites (glucose, GLU; total protein, TP); intermediate metabolites (creatinine; total bilirubin, TBil; total cholesterol TC; urea); enzymes (alkaline phosphatase, ALP; alanine aminotransferase, ALT; aspartate aminotransferase, AST; creatine phosphokinase, CPK; gamma-glutamyl transferase, γ-GT; lactate dehydrogenase, LDH); macro- and micro-elements (calcium, Ca; chloride, Cl; copper, Cu; iron, Fe; magnesium, Mg; phosphorus, P; potassium, K; selenium, Se; sodium, Na; zinc, Zn). Biochemical parameters obtained were compared to those reported by Kaneko and co-authors [23] and Latimer and co-authors [24] for reference values of the species. Plasmatic concentration of α-TOH was determined by high-pressure liquid chromatography coupled with an ultraviolet detector (HPLC-UV). All standards and solvents were purchased from Sigma Aldrich (Milan, Italy). Stock solution (1 mg/ mL) of α-TOH was prepared in chloroform/methanol (50/50). For the calibration curve, standard stock solutions were diluted with methanol and kept frozen at − 20 °C, protected from light. Plasma levels of α-TOH were measured at 280 nm.

Chromatographic separation was carried out on a Waters Symmetry C18 column (4.6 × 150 mm, particle size 5 μm, Waters, Milford, MA). The injection volume was 20 μL. The mobile phases used were acetonitrile/methanol/Milli-Q water (64.5/33/2.5) at 1 mL/min. Data were acquired and processed by Breeze Software (Waters, Milford, MA). Samples were prepared as follows: 0.3 mL of serum was vortexed with 0.6 mL of acetonitrile and centrifuged at 3500*g* at 4 °C for 10 min. The supernatant was dried under a stream of nitrogen and the residue was reconstituted in 0.15 mL of the mobile phase [2, 17, 25].

The aliquot destined to Cu, Se and Zn determination underwent the total mineralization. For the purpose, 0.5 g of plasma was weighed into a microwave digestion vessel with 8 mL of HNO_3_ 65% and 2 mL of H_2_O_2_ 30% and decomposed by microwave digestion using Ethos Easy (Milestone s.r.l., Sheldon, CT, USA.). On each aliquot, 20% was prepared in duplicate. The whole process was monitored thanks to two samples of Animal Blood Reference Material (IAEA- A- 13) and analysed with experimental samples. The digested solution, properly diluted, was analysed using an ICP-MS spectrometer model NexlON 300 X equipped with an autosampler model S10 (PerkinElmer, Monza, Italy).

The optimized parameters used during ICP-MS measurements were: RF generator power output: 1600 W; argon flows; plasma, 17.993 dm^3^ min^−1^; nebulizer, 0.991 dm^3^ min^−1^; auxiliary 1.203 dm^3^ min^−1^; He flow: 3.5 cm^3^ min^−1^. Quantification was performed using external calibration with a 1 mg dm^−3^ Rh solution as an internal standard.

The external calibration line was obtained on three different concentration levels (*R*^2^ > 0.999). The recovery of CRM was 99%.

Individual semen samples were collected through an artificial vagina (Colorado) at the stud centre of the Autonomous Region of Sardinia. Semen samples were analysed in the laboratory of the Department for Research in Equine Reproduction of the Autonomous Region of Sardinia. Exclusively for semen samples, collection was carried out over the three last days of *T*_1_ to minimize the time effect on immobile spermatozoa from random sampling. After filtration, each gel-free ejaculate was immediately checked for pH determination and colour inspected (to exclude abnormal presence of blood in the trace). Subsequently, quantitative analysis considered sperm cell concentration (million/mL) and morphology, motility (percentage of mobile and immobile sperm cells) and integrity. Additionally, among mobile percentage also those progressive were determined and the straightness index calculated. All parameters were obtained and analysed by a semiautomatic digital equipment and data analysed by the Microptic Sperm Class Analyzer® CASA System. Values were determined within 0, at 24 and 48 h of sampling and values averaged, following the protocol described before. Data were mediated over the three aliquots of semen samples for each horse involved in the trial.

### Analysis of Data and Statistical Methods

Values of investigated parameters were computed for each horse.

Data were analysed by using the following statistical model, according to a mixed procedure of the analysis of variance:

$$ {Y}_{i,j,k}=\mu +{D}_{i,j}+{G}_{j,k}+{D}_{i,j}\ast {G}_{j,k}+{e}_{i,j,k} $$

where *Y* is the dependent variable (haematological parameters; biochemical parameters; semen traits), *μ* is the overall mean, *D* is the fixed effect of the diet (two levels: CON vs. SUPP100), *G* is the fixed effect of age (two levels: below or equal vs. above median age of 13 years) and D * G is the interaction factor. The animal was the random effect, whereas plasmatic levels of α-TOH, Cu, Se and Zn were used as co-variates.

Confidence intervals and grouping were adjusted according to Tukey method. All data were analysed using SAS 9.2 (SAS Inst. Inc. Cary, NC). Statistical significance was set for *p* value < 0.05.

## Results

All animals enrolled in the trial appeared healthy at start (Table 2) and throughout the experimental period. However, the biochemical analysis of *T*_0_ blood samples pointed to a deficient condition of circulating α-TOH and Zn (Table 2). Stallions of the supplemented group ingested 204 ± 10.4 mg/100 kg BW/day of all-rac-tocopheryl-acetate, representing 2.5-fold of daily intake of non-supplemented control stallions and additional Se-meth to sodium-selenite of the compound feed. Plasmatic levels of α-TOH (1.21 ± 0.05 μg/mL vs. 2.11 ± 0.11 μg/mL, *p* < 0.0001), Zn (535 ± 188 vs. 789 ± 120 μg/L, *p* = 0.031) and total Se (119 ± 10.4 vs. 236 ± 37.4 μg/L, *p* = 0.027) differed significantly between groups at the end of the feeding trial.

**Table 2:** Serum biochemical profile and plasmatic levels of Zn, Cu, Se and α-TOH determined in the totality of stallions at the beginning of the trial, as allotted to the two experimental groups following the matched-pairs approach

Item^2^	Reference range^3^	Diet^1^		Pooled SD	*p* value^5^
		CON	SUPPL100		
T P (g/L)	52.0–79.0	71.3	72.5	0.54	0.406
ALB (g/L)	26.0–41.0	40.8	40.9	0.34	0.964
UREA (mg/dL)	11.0–27.0	24.6	26.2	4.40	0.272
CREA(mg/dL)	0.40–2.20	1.54	1.49	0.26	0.501
AST (U/L)	160–412	264	268	44.3	0.966
ALT (U/L)	3.00–23.0	20.5	17.5	9.49	0.316
γ-GT (U/L)	6.00–32.0	25.8	26.3	4.01	0.696
TB (mg/dL)	0.00–3.20	1.84	2.03	0.57	0.300
ALP (U/L)	143–359	163	162	33.7	0.944
Zn (μg/L) p	600–1200	590	560	212	0.794
Cu (μg/L) p	500–1500	1110	1040	274	0.900
Se (μg/L) p	100–250	121	120	49.9	0.978
α-TOH (μg/mL) p	1.50–2.00^4^	0.87	0.86	0.34	0.808

^1^Diet: *CON*, control diet; *SUPPL100*, diet containing 100 g/day per horse of supplement

^2^Item: *ALB*, albumins; *CREA*, creatinine; *AST*, aspartate aminotransferase; *ALT*, alanine aminotransferase; *γ-GT*, gamma-glutamyl aminotransferase; *TB*, total bilirubin; *ALP*, alkaline phosphatase; *α-TOH*, alpha-tocopherol; *p*, plasmatic

^3^Reference value: according to Kaneko et al. [23] and Latimer et al. [24]

^4^Reference value: Muirhead et al. [30]

^5^*p* value: *p* < 0.05 indicates a significant effect of linear and/or quadratic contrasts

No differences are pointed out between the metabolic profile of horses allotted to the two dietary treatments at start. All the horses displayed to possess low circulating values of Zn and α-TOH, without clinical signs of specific deficiency

The haematological profile of horses pointed to differences between groups as reported in Table 3. Levels of basophils (BASO) and mean cell haemoglobin concentration (MCHC) turned out to differ according to the dietary group (*p* = 0.014 and *p* = 0.030, respectively), being both lower in the supplemented group. At the interaction of levels of circulating Cu, Se and Zn (Table 3), a frequent effect of Se levels per se and of Zn levels x the diet were observed on circulating neutrophils (NEU), monocytes (MONO) and eosinophils (EOS).

**Table 3:** Haemogram of stallions fed with the two dietary treatments at the end of the experimental feeding (*T*_1_)

Item^2^	Reference range^3^	Diet^1^		Pooled SD	*p* value^4^						
		CON	SUPPL100		Diet	Age class	α-TOH levels	Cu levels	Zn levels	Se levels	Animal
WBC (10^9/L)	4.90–10.3	7.65	7.15	1.34	0.409	0.823	0.561	0.737	0.249	0.770	0.062
LYMPH (10^9/L)	2.20–8.10	2.50	2.37	0.72	0.946	0.808	0.957	0.699	0.908	0.134	0.035
NEU (10^9/L)	1.70–5.80	4.31	4.12	1.03	0.345	0.506	0.214	0.784	0.033	0.133	0.500
MONO (10^9/L)	0.00–1.52	0.60	0.45	0.42	0.549	0.259	0.079	0.088	0.313	0.022	0.876
EOS (10^9/L)	0.00–0.80	0.19	0.17	0.06	0.874	0.605	0.953	0.769	0.858	0.057	0.390
BASO (10^9/L)	0.00–0.30	0.05	0.03	0.02	0.014	0.330	0.773	0.817	0.143	0.648	0.265
RBC (10^12/L)	6.20–10.2	8.77	8.21	1.33	0.876	0.574	0.873	0.831	0.209	0.096	0.112
HGB (g/L)	63.0–132	151	143	23.0	0.983	0.716	0.837	0.866	0.268	0.085	0.139
HCT (%)	31.0–50.0	39.7	38.0	6.23	0.976	0.993	0.638	0.820	0.303	0.022	0.187
MCV (fL)	37.0–53.0	45.4	46.4	2.69	0.456	0.025	0.567	0.770	0.460	0.013	0.196
MCH (pg)	14.0–20.0	17.3	17.5	0.77	0.741	0.370	0.707	0.666	0.885	0.773	0.272
MCHC (g/L)	360 – 390	382	378	16.3	0.030	0.196	0.237	0.822	0.568	0.010	0.348

^1^Diet: *CON*, control diet; *SUPPL100*, diet containing 100 g/day per horse of supplement

^2^Item: *WBC*, white blood cell; *LYMPH*, lymphocyte; *NEU*, neutrophil granulocytes; *MON*, monocytes; *EOS*, eosinophil granulocytes; *BASO*, basophil granulocytes; *RBC*, red blood cell; *HGB*, haemoglobin; *HCT*, haematocrit; *MCV*, mean cell volume; *MCH*, mean cell haemoglobin; *MCHC*, mean cell haemoglobin concentration

^3^Reference value according to Kaneko et al. [23] and Latimer et al. [24]

^4^*p* value: *p* < 0.05 indicates significant effect of linear and/or quadratic contrasts

**Fig. 1:**
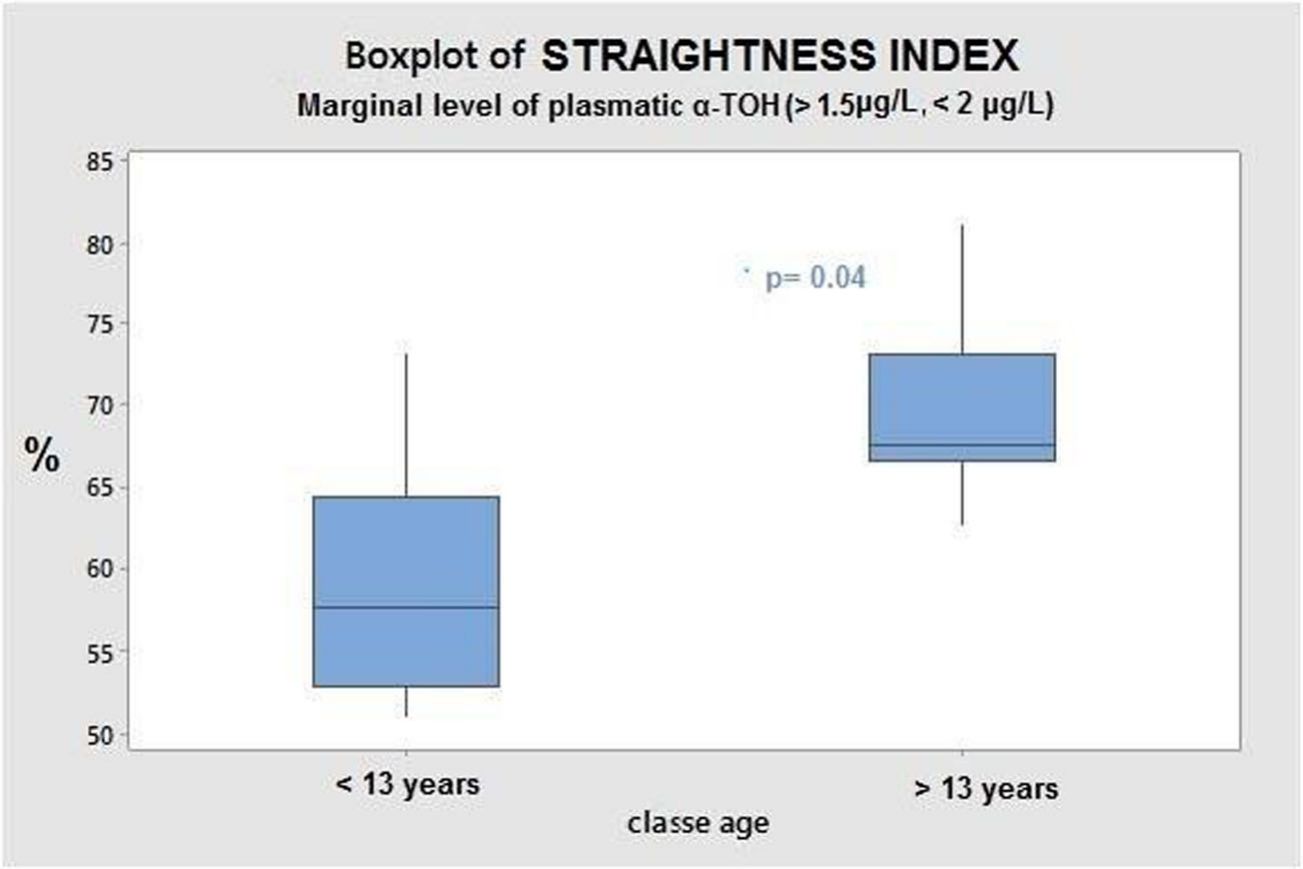
The boxplot displays the average percentage of normal and mobile sperm cells with straight movement determined for stallions after the dietary supplementation. Results are referred to as horses with marginal levels of α-TOH and re-established values of plasmatic Zn and Se and in the background of adequate circulating Cu levels. Significant difference was established for *p* value< 0.05

With regard to semen quality, no differences could be pointed out as to concentration of sperm cells, percentage of immobile and percentage of mobile x progressive spermatozoa between the two groups (Table 4). However, the significant effect of the individual animal was found as to the straightness index (*p* = 0.032). Independently on supplementation, the interaction with age did not lead to differences in semen traits of stallions of the two groups, except for the percentage of sperm cells with straight mobile activity in stallions with age > 13 years and adequate circulating α-TOH (*p* = 0.004), in view of re-established plasmatic values of Zn, Se, with a background of adequate circulating Cu (see graph in Fig. 1). The pH-value in semen samples of both groups did not point to differences, ranging between 7.0 and 7.5. The metabolic profile of horses of *T*_1_ samples was explored according to the different levels of circulating α-TOH, on the basis of low, marginal and adequate levels obtained through the modulator effect of the diet (Table 5).

**Table 4:** Analysed semen traits of stallions fed with the two dietary treatments at the end of *T*_1_ phase

Sperm cell	Diets^1^		Pooled SD	*p* value^2^						
	CON	SUPPL100		Diet	Age class	α-TOH levels	Cu levels	Zn levels	Se levels	Animal
Concentration (Mil/mL)	386	284	259	0.594	0.374	0.886	0.922	0.839	0.273	0.413
Immobile (%)	28.6	22.4	18.4	0.806	0.666	0.249	0.265	0.216	0.654	0.383
Mobile*progressive (%)	40.9	44.1	18.2	0.658	0.156	0.336	0.203	0.240	0.616	0.067
Straightness index	66.2	65.7	7.80	0.882	0.266	0.613	0.360	0.430	0.768	0.032

^1^Diet: *CON*, control diet; *SUPPL100*, diet containing 100 g/day per horse of supplement

^2^*p* value: *p* < 0.05 indicates a significant effect of linear and/or quadratic contrasts

**Table 5:** Biochemical profile of stallions according to α-TOH status at the end of *T*_1_ phase

Item^2^	Reference range^3^	Plasmatic α-TOH levels^1^			Pooled SD	*p* value
		Low	Marginal	Adequate		
TP (g/L)	52.0–79.0	52.2	65.3	77.1	21.1	0.012
UREA (mg/dL)	26.0–41.0	17.5	28.8	23.5	13.7	0.047
ALT (U/L)	10.0–24.0	6.46	6.23	5.87	3.67	0.918
AST (U/L)	226–366	197	224	269	77.3	0.071
γ-GT (U/L)	6.00–32.0	11.2	14.3	17.9	5.72	0.014
TBil (mg/dL)	0.00–3.20	2.04	3.08	2.91	1.14	0.018
ALP (U/L)	143–359	106	140	171	45.7	0.002
TC (mmol/L)	1.43–3.59	1.91	2.15	2.46	0.80	0.208
Ca (mmol/L)	2.80–3.40	2.44	2.94	3.26	0.74	0.017
P (mmol/L)	1.00–1.18	0.81	1.07	0.98	0.28	0.021
Mg (mmol/L)	0.90–1.15	0.61	0.78	0.83	0.22	0.015
Na (mmol/L)	136–142	170	169	168	1.18	0.002
Chloride (mmol/L)	99.0–109	84	100	101	14.5	0.001
K (mmol/L)	2.40–5.20	4.52	4.14	3.90	2.15	0.766
Fe (μmol/L)	13.0–37.0	27.9	39.4	41.9	10.5	0.003
Zn (μg/L)	600–1200	480	577	820	288	0.017
Cu (μg/L)	500–1500	1220	718	1010	758	0.137
Se (μg/L)	100–250	79.2	183	281	33.1	0.001
LDH (U/L)	112–456	242	258	291	86.6	0.365
Glu (mmol/L)	3.50–5.90	3.54	5.18	4.99	1.62	0.005

^1^Plasmatic α-TOH levels: Low = < 1.5 μg/mL; Marginal = > 1.5 and < 2 μg/mL; adequate= > 2 μg/mL

^2^Item: *TP*, total protein; *ALB*, albumins; *ALT*, alanine aminotransferase; *AST*, aspartate aminotransferase; *ALP*, alkaline phosphatase; *TC*, total cholesterol; *LDH*, lactic dehydrogenase

^3^Reference value: according to Kaneko et al. [23] and Latimer et al. [24]

## Discussion

Common feeding practices of breeding stallions are based on hay and compound feed administered to the horse individually housed in the box. All the stallions screened in this study showed deficient plasmatic levels of α-TOH. Plasmatic levels of Se instead were within the reference range for the horse, likewise those of plasmatic Cu. However, circulating Zn levels appeared below the adequate reference range. It is noteworthy to point out that the interactions between such elements (Cu, Se and Zn) together with the plasmatic levels of α-TOH may provide sufficient metabolic milieu to avoid the onset of clinical disturbances associated with the specific deficiency, like reported by other authors [43]. In fact, the same authors state that despite many horses had very low Zn and Se intakes, no signs of deficiencies were present in the study population concluding that at least in horses with adequate Vit E intake recommendations of those elements may be overestimated. No clinical signs could also be found in equines fed on pastures (ideally consuming large amounts of natural α-TOH from vegetation), in view of adequate circulating elements [5, 6] The hypothesis that dietary antioxidants could have an effect in improving semen traits of breeding stallions was tested in this trial under different metabolic and clinical aspects. For this purpose, stallions underwent an 8-week feeding trial, during the negative photoperiod, 4 months before entering the new stud season. The choice of the length of the feeding trial was aligned to the protocols adopted in the stud centre to evaluate health conditions of horses as well as semen characteristics. In addition, the stallion is susceptible to photoperiod, though to a lower extent than the mare. Semen production appears to decrease as natural light hours also decrease [40]. That way, the negative photoperiod could be helpful to maximize the effects of dietary supplementation.

The interaction between nutrients with antioxidant effect and the metabolic status of stallions enrolled was a *condition* sine qua non for the correct interpretation of the biological effect exerted by the diet. The metabolic profile of SUPPL100 stallions did not point to any deficiency in plasmatic levels of target elements (Cu, Se and Zn) nor in circulating α-TOH. The re-establishment of adequate levels of plasmatic Zn was observed in all supplemented stallions and this is a key factor in the improvement of the metabolic status of horses. However, it is to underline that Se and Cu resulted to be appropriate in stallions at start. The synergy between Zn, Cu and Se (enrolled in different antioxidant endogenous systems) allows to reckon that probably no specific deficiency was found due to the complementary biological role of those elements with antioxidant roles of α-TOH. At this regard, Cu is actively engaged in cytochrome oxydase and Cu-Zn superoxide dismutase (SOD) for cell respiration and antioxidant roles. Moreover, Se is a component of the glutathione peroxidase (GPX) in the cytosol of different cell types, including blood cells, which effects were also seen in the variation of haematological profile of stallions after supplementation. Moreover, Se is involved in several biochemical systems to prevent lipid peroxidation and in particular for sperm cells, it is a constituent of sperm capsule selenoprotein.

On quantitative assessment and microscopic analysis of semen traits, particular attention was paid to the proportion between mobile and immobile spermatozoa percentages, which may affect the chance for successful conception. As to mobile spermatozoa, the “straightness” parameter is associated with a higher chance for progressive and linear movement. In this regard, the computer-assisted sperm analysis (CASA) allowed to test the movement of spermatozoa from fresh semen samples [9, 11, 34], following the methods already tested in the literature. Of note, the high metabolic activity of spermatozoa and energy costs in terms of survival rate outside male reproduction system can reasonably allow to expect abundance of reactive oxygen species (ROS) in the cell. Arguably, lipid peroxidation may contribute to sperm cell membrane damage and dramatically reduce fertility potentials. The oxidative stress may consequently impair spermatozoa vitality, in which unsaturated fatty acid content in cytoplasm is naturally high. Recently, Finno and Valberg [15] reviewed the biological activities in different body systems in which vitamin E takes part. The higher circulating level of α-TOH observed in the totality of SUPPL100 stallions in comparison to that determined in CON horses appears encouraging and sheds a new light on the interpretation of results, in view of reference literature. The lipophilic property of α-TOH appears of systemic importance for stability and protection of cell membrane at different levels (circulating cells in the bloodstream as well), in agreement with observation earlier reported against ROS at systemic level [33]. Circulating levels in the bloodstream determined in the serum of horses at start turned out to be deficient (~ 1 μg/mL) [30, 36]. The natural source of α-TOH in the diet of the horse is represented by fresh fodder whereas its content progressively diminishes in hay, due to thermo- and photolability of this fat-soluble vitamin. In view of this fact, the introduction of α-TOH is of crucial importance in the diet of the stallion, commonly housed in individual boxes and fed on hay and compound feeding stuffs. On the other hand, pasture contamination with agents showing a direct effect on semen volume is often overlooked [1, 14], but clinical cases on breeding stallions are not reported, to the best of our knowledge. Systemic and local oxidative stress may be augmented during the stud season and semen production may be affected if dietary formulations do not meet nutritional requirements. According to circulating levels of α-TOH (below or above 2 μg/mL) determined in horses involved in this experimental feeding trial, the analysed traits to assess semen quality did not show significant differences. These findings are in agreement with those reported by other authors [10]. Our results on semen traits following the dietary effects are in contrast with other works reported in the literature, in which, however, the metabolic status of horses was not explored in details like in this investigation [3, 9, 10, 37].

In this trial, a systemic metabolic approach turned out to be useful to explain the effects of supplementation. In fact, in light of the overall metabolic status and circulating levels of target biochemical parameters, the interpretation of the results on semen traits was helpful to identify deficiencies.

In view of the overall systemic evaluation, it must be said that probably the duration of the experimental supplementation (8 weeks) is short if compared to the physiological intervals of spermatogenesis, taking altogether spermatocytogenesis, meiosis and spermiogenesis which lasts 57 days in the stallions [22]. However, bearing in mind that both blood and sperm samples are heterogeneous tissues in terms of cell age, the limiting effect from the duration of the trial can reasonably be considered as low impacting. In the large part of stallions enrolled in the trial (older than 13 years of age) the marginal level of circulating α-TOH was achieved after dietary supplementation. In those animals, a significantly higher percentage of straightness in normal and motile sperm cells was observed.

Supplementation of α-TOH, Zn and Se-meth at tested amount in the diet of breeding stallions (following the preliminary assessment of circulating micronutrient) helped to define plasmatic levels of biochemical parameters and respective potential interactions at systemic level. The re-establishment of adequate levels of plasmatic α-TOH, Cu, Se and Zn as antioxidants helped to evaluate the need for supplementation in case of deficient conditions, important to prevent the onset disturbances in case of augmented activity, like during the stud season. The systemic metabolic and haematological profile of stallions was ameliorated with respect of the dietary supplementation. Haemogram was also improved after supplementation being horses at start deficient is plasmatic Zn and with marginal levels of Se. In particular, the diet effect produced lower circulating BASO in the supplemented stallions, though being within the physiological range for both groups. In this regard, BASO are polymorphonuclear cells with cytoplasmic enzyme-containing granules showing a high affinity to basic histological staining dyes. They are involved in different roles of the immune response, in particular by modulating or participating to differentiation of T-helper type 2 lymphocytes. BASO are mobile cells and can be recruited by tissues, thus leaving the bloodstream [7]. The increase of BASO in the bloodstream may indicate that inflammation is occurring in body tissues and that BASO are required. In this trial, the significant reduction of BASO may be interpreted as a lower inflammatory status, but this hypothesis was not investigated further. Rather, all stallions monitored during the trial appeared clinically healthy and this is in agreement with the haemograms found, independently of the dietary treatment. In addition, MCHC was found to vary significantly in horses, according to the diet. Indeed, the concentration of haemoglobin (HGB) within RBC is depending on the fact that also HGB was found lower in supplemented stallions, though not significantly. This finding deserves to be investigated further, in view of the potential interactions between micro-elements and the martial cycle for HGB re-synthesis. However, the dietary effect on semen traits was not consistently found, except for the case when marginal plasmatic levels of α-TOH were achieved in stallions older than 13 years. The straightness index of sperm cells also improved, in the background of re-established Zn and Cu circulating levels. In conclusion, results appear encouraging to pave the way to test the dietary supplementation in respect of the physiological stage and need of stud stallions also on the long-term period.

## References

[1] Aboling S, Drotleff AM, Cappai MG, Kamphues J (2016). Contamination with ergot bodies (*Claviceps purpurea sensu lato*) of two horse pastures in Northern Germany. Mycotoxin Res.

[2] Biesalski H, Greiff H, Brodda K, Hafner G, Bässler KH (1986). Rapid determination of vitamin A (retinol) and vitamin E (alpha-tocopherol) in human serum by isocratic adsorption HPLC. Int J Vitam Nutr Res.

[3] Brinsko SP, Varner DD, Love CC, Blanchard TL, Day BC, Wilson ME (2005). Effect of feeding a DHA-enriched nutriceutical on the quality of fresh, cooled and frozen stallion semen. Theriogenology.

[4] Burk RF, Nosworthy BK, Hill KE, Motley AK, Byrne DW (2006). Effects of chemical form of selenium on plasma biomarkers in a high-dose human supplementation trial. Cancer Epidemiol Biomark Prev.

[5] Cappai MG, Dimauro C, Biggio GP, Cherchi R, Accioni F, Pudda F, Boatto G, Pinna W (2020). The metabolic profile of Asinara (albino) and Sardo donkeys (pigmented) (Equus asinus L., 1758) points to unequivocal breed assignment of individuals. PeerJ.

[6] Cappai MG, Pudda F, Wolf P, Accioni F, Boatto G, Pinna W (2020). Variation of hematochemical profile and vitamin E status in feral Giara horses from free grazing in the wild to hay feeding during captivity. J Equine Vet Sci.

[7] Chirumbolo S (2012). State-of-the-art review about basophil research in immunology and allergy: is the time right to treat these cells with the respect they deserve?. Blood Transfus.

[8] Colembrander B, Gadella BM, Stout TAE (2003). The predictive value of semen analysis in the evaluation of stallion fertility. Reprod Domest Anim.

[9] Contri A, De Amicis I, Molinari A, Faustini M, Gramenzi A, Robbe D, Carluccio A (2011). Effect of dietary antioxidant supplementation on fresh semen quality in stallion. Theriogenology.

[10] Deichsel K, Palm F, Koblischke P, Budik S, Aurich C (2008). Effect of dietary antioxidant supplementation on semen quality in pony stallions. Theriogenology.

[11] Estrada AJ, Samper JC, Samper JC, Pycock JF, McKinnon AO (2006). Evaluation of raw semen. Current therapy in equine reproduction.

[12] Evans HM, Bishop KS (1922). On the of the hitherto unrecognized dietary factor essential for reproduction. Science.

[13] Fairweather-Tait SJ, Collings R, Hurst R (2010). Selenium bioavailability: current knowledge and future research requirements. Am J Clin Nutr.

[14] Fayrer-Hosken RA, Hill NS, Heusner GL, Traylor-Wiggins W, Turner K (2013). The effects of ergot alkaloids on the breeding stallion reproductive system. Equine Vet J Suppl.

[15] Finno CJ, Valberg SJ (2012). A comparative review of vitamin E and associated equine disorders. J Vet Intern Med.

[16] Fordyce FM, Selinus O (2013). Selenium deficiency and toxicity in the environment. Essentials of medical, Geology edn.

[17] Ganiére-Monteil C, Kergueris MF, Pineau A, Blanchard B, Azoulay C, Larousse C (1994). Determination of plasma retinol and alpha-tocopherol by HPLC. Ann Biol Clin.

[18] Grace ND, Pearce SG, Firth EC, Fennessy PF (1999). Content and distribution of macro- and micro-elements in the body of pasture-fed young horses. Aust Vet J.

[19] Haygarth PM, Cooke AI, Jones KC (1993). Long-term change in the biogeochemical cycling of atmospheric selenium: deposition to plants and soil. Geophys Res.

[20] Henneke DR, Potter GD, Kreider JL, Yeates BF (1983). Relationship between body condition score, physical measurements and body fat percentage in mares. Equine Vet J.

[21] Jasko DJ, Lein DH, Foote RH (1990). Determination of the relationship between sperm morphologic classifications and fertility in stallions: 66 cases (1987-88). J Am Vet Med Assoc.

[22] Johnson L, Blanchard TL, Varner DD, Scrutchfield WL (1997). Factor affecting spermatogenesis in the stallion. Theriogenology.

[23] Kaneko JJ, Harvey JW, Bruss ML (2008). Clinical biochemistry of domestic animals. VI Edn.

[24] Latimer KS (2011). Duncan & Prasse’s veterinary laboratory medicine: clinical pathology. V Edn.

[25] Levent A, Oto G, Ekin S, Berber I (2013). Method validation and simultaneous determination of retinol, retinyl palmitate, β-carotene, α-tocopherol and vitamin C in rat serum treated with 7,12 dimethylbenz[a]anthracene and *Plantago major* L. by high- performance liquid chromatography using diode-array detection. Comb Chem High Throughput Screen.

[26] Love CC, Varner DD, Thompson JA (2000). Intra and inter-stallion variation in sperm morphology and their relationship with fertility. J Reprod Fertil Suppl.

[27] Mehdj Y, Hornick JL, Istasse L, Dufrasne I (2013). Selenium in the environment, metabolism and involvement in body functions. Molecules.

[28] Meyer H, Ahlswede L (1976). Über das intrauterine Wachstum und Körperzusammensetzung von Fohlen der Nährstoffbedarf tragenden Stuten. Ubersich Tierernahr.

[29] Morris LHA, Allen WR (2002). Reproductive efficiency of intensively managed thoroughbred mares in Newmarket. Equine Vet J.

[30] Muirhead TL, Witchel JJ, Stryhn H, McClure JT (2010). The selenium and vitamin E status of horses in Prince Edward Island. Can Vet J.

[31] National Research Council (NRC) (2007). Nutrient requirements of Horses.

[32] Naumann C, Bassler R (2004). Chemical analysis of animal feed.

[33] Naziroğlu M, Karaoğlu A, Orhan A (2004). Selenium and high dose vitamin E administration protects cisplatin-induced oxidative damage to renal, liver and lens tissues in rats. Toxicology.

[34] Nervo T, Semita C, Pescarolo C (2010). Analisi computerizzata di seme equino fresco e refrigerato appartenente a tre razze differenti. Ippologia.

[35] Picket BW (1993) Factors affecting sperm production and output. In: McKinnon AO and Voss JL (Eds), Equine Reproduction 78, 689–704. Philadelphia

[36] Pusterla N, Puschner B, Steidl S, Collier J, Kane E, Stuart RL (2010). Alfa-tocopherol concentration in equine serum and cerebrospinal fluid after vitamin E supplementation. Vet Rec.

[37] Schmid-Lausigk Y, Aurich C (2014). Influences of a diet supplemented with linseed oil and antioxidants on quality of equine semen after cooling and cryopreservation during winter. Theriogenology.

[38] Sullivan JJ, Turner PC, Self LC, Gutteridge HB, Bartlett DE (1975). Survey of reproductive efficiency in the Quarterhorse and thoroughbred. J Reprod Fertil Suppl.

[39] Tapiero H, Townsend DM, Tew KD (2003). The antioxidant role of selenium and seleno-compounds. Biomed Pharmacother.

[40] Taras A (2013) Valutazione pluriennale delle caratteristiche riproduttive in stalloni impiegati in un programma di monitoraggio del materiale seminale. Ph D Thesis, University of Sassari; Italy

[41] Van Buiten A, Van den Broek J, Schukken YH, Colebrander B (1999). Validation of no-return rate as a parameter for stallion fertility. Livest Prod Sci.

[42] Van Soest PJ, Robertson JB, Lewis BA (1991). Methods for dietary fiber, neutral detergent fiber and non starch polysaccharide in relation to animal nutrition. J Dairy Sci.

[43] Wichert B, Frank T, Kienzle E (2002). Zinc, copper and selenium intake and status of horses in Bavaria. J Nutr.

[44] Zakošek Pipanm M, Mrkun J, Nemec Svete A, Zrimšek P (2017). Improvement of liquid stored boar semen quality by removing low molecular weight protein and supplementation with α-tocopherol. Anim Reprod Sci.

